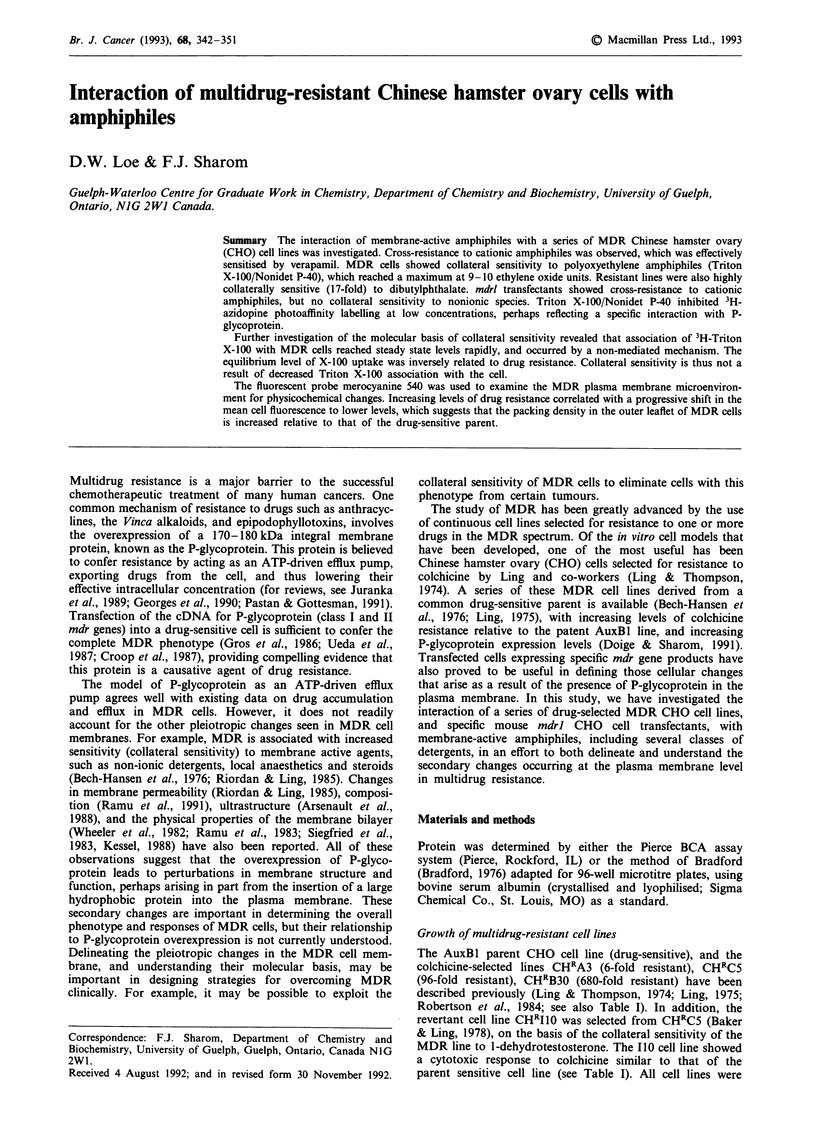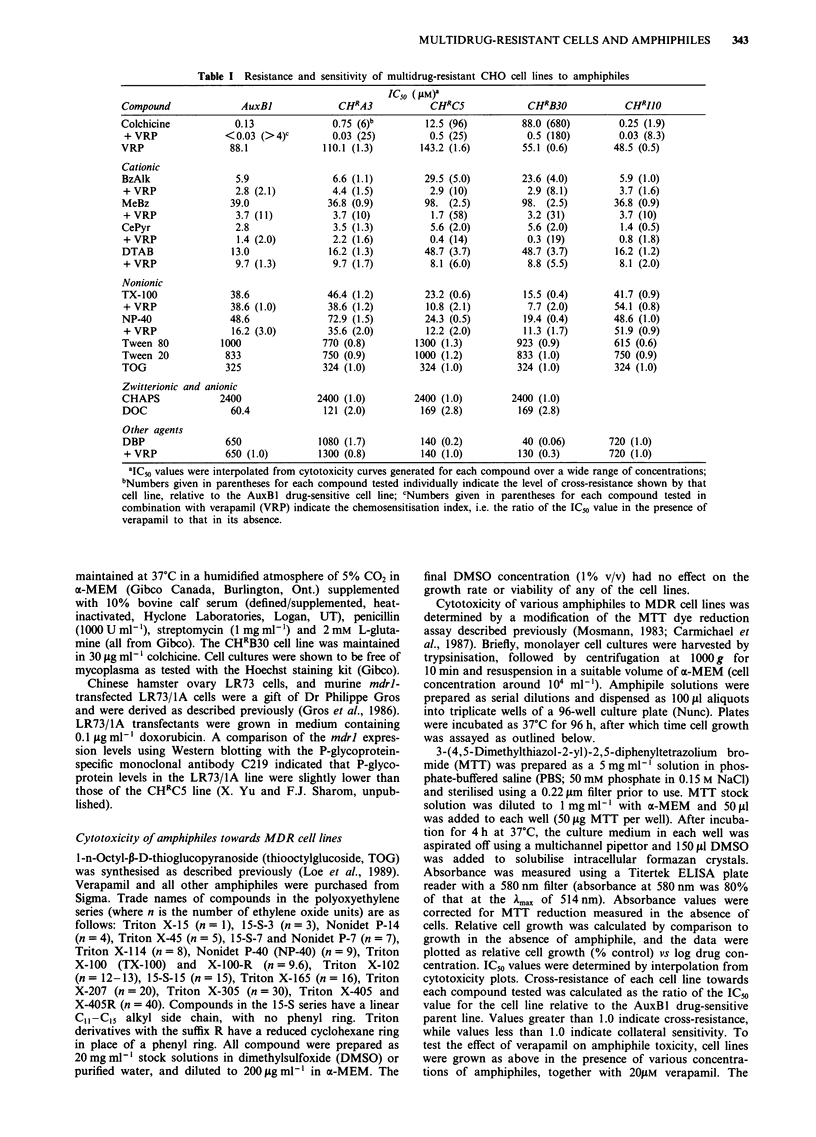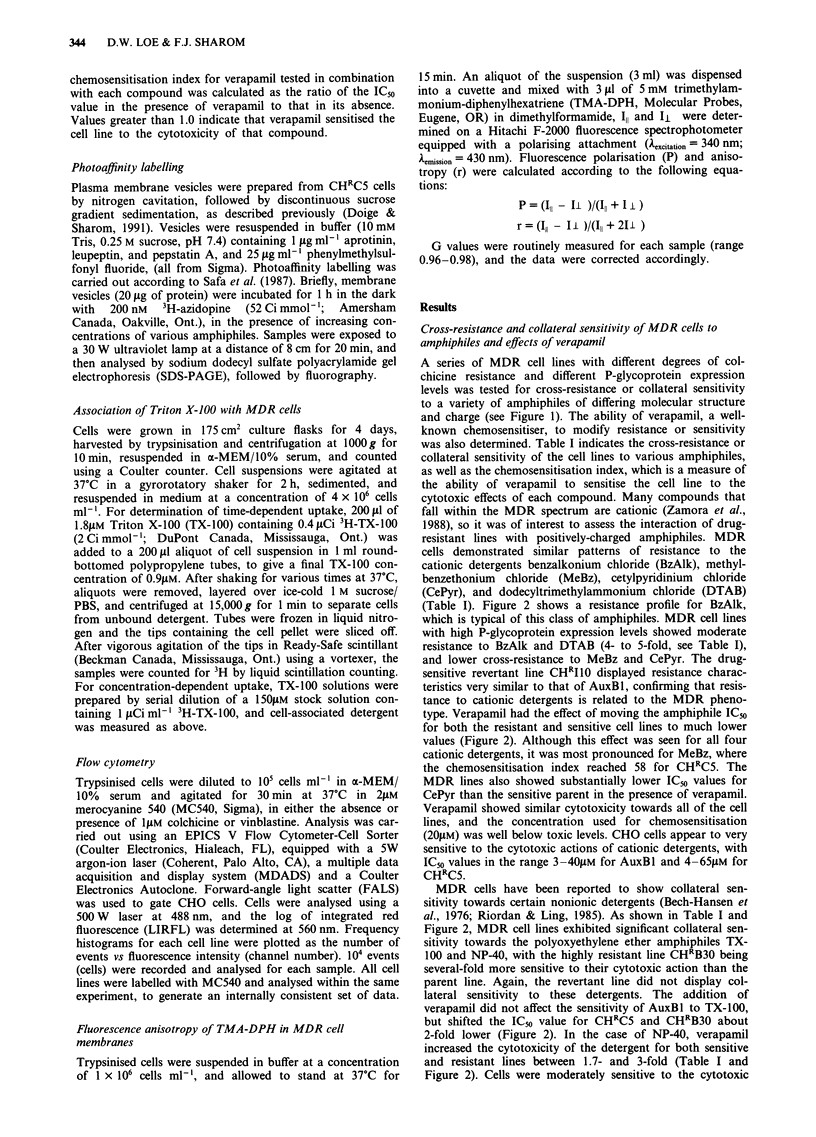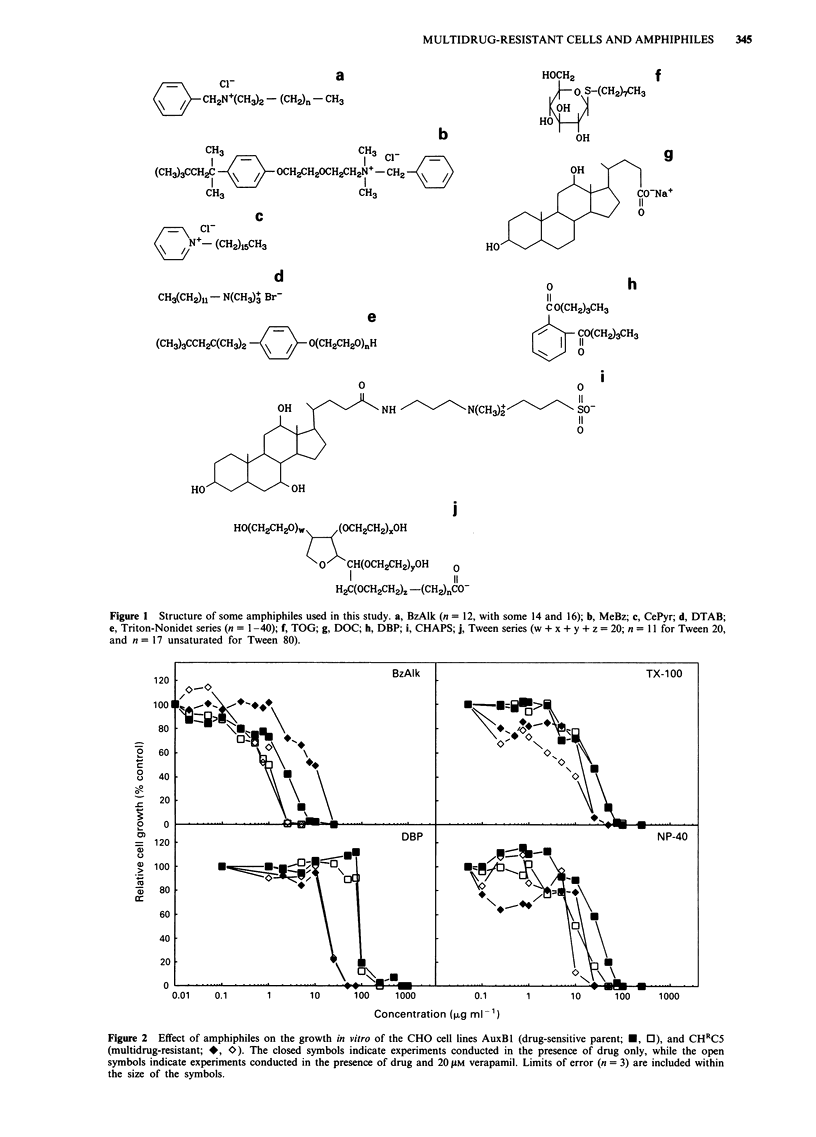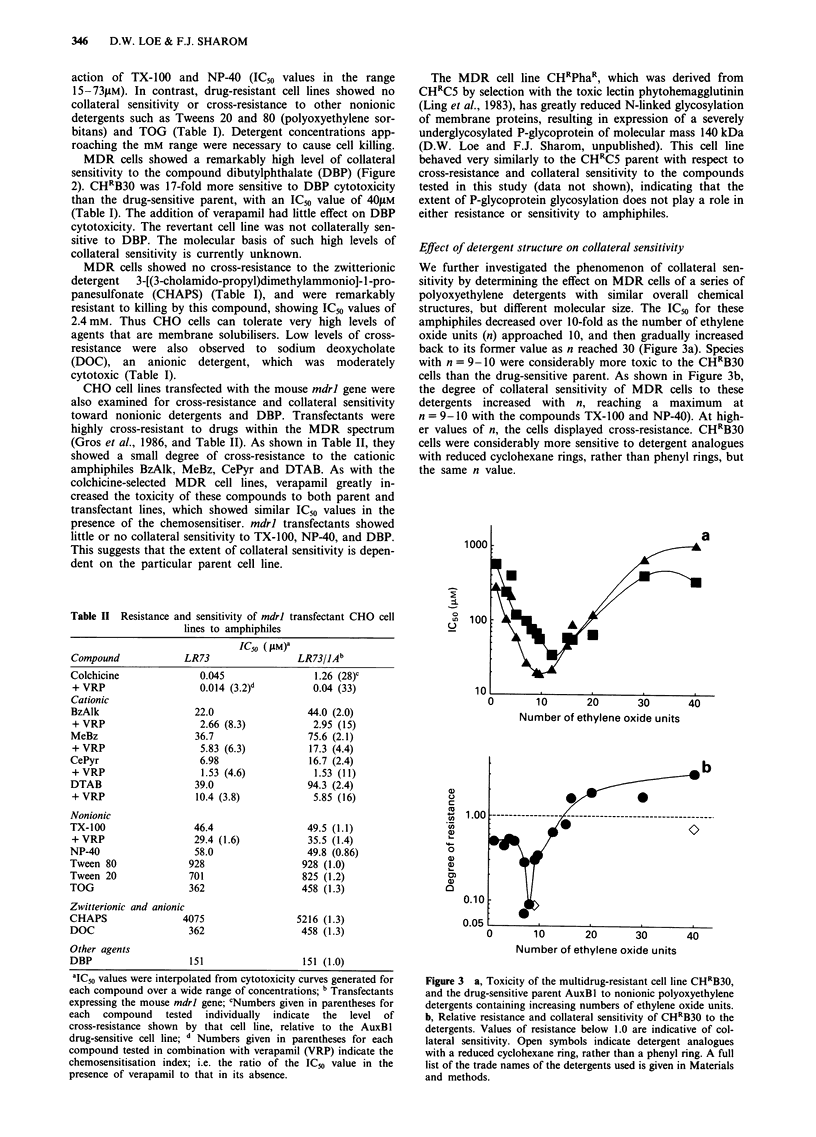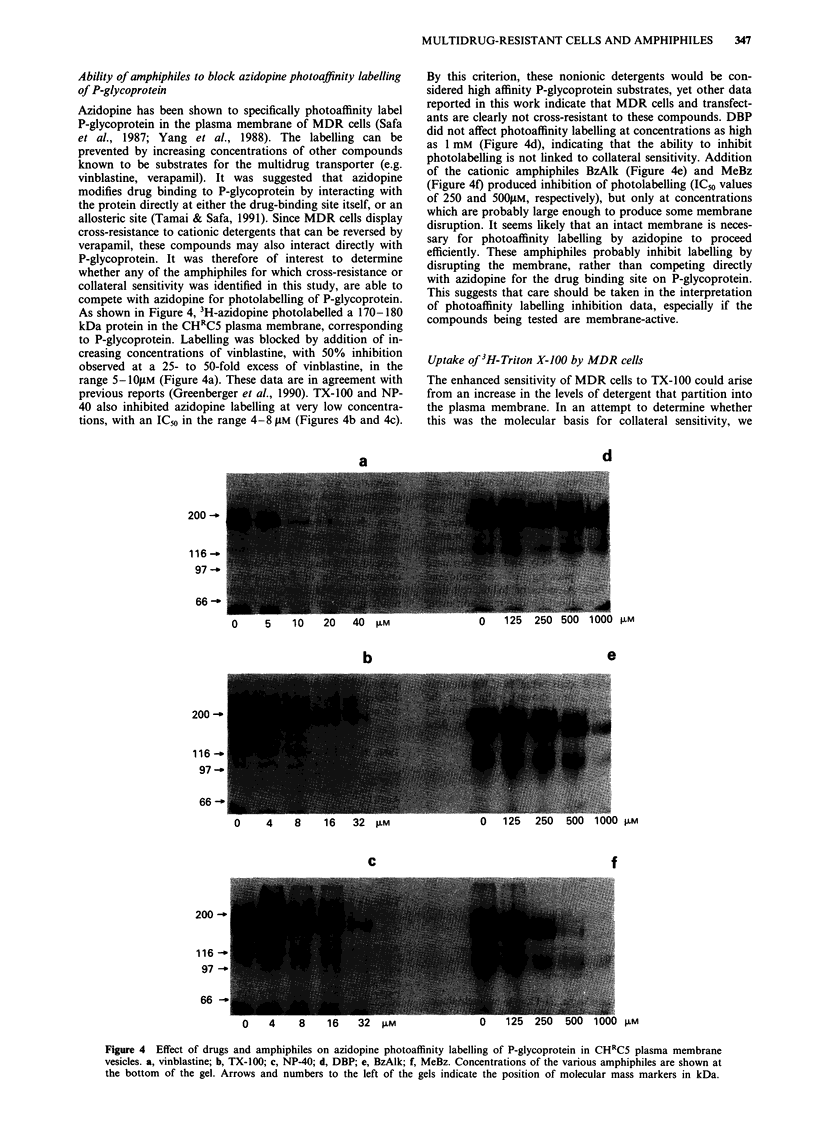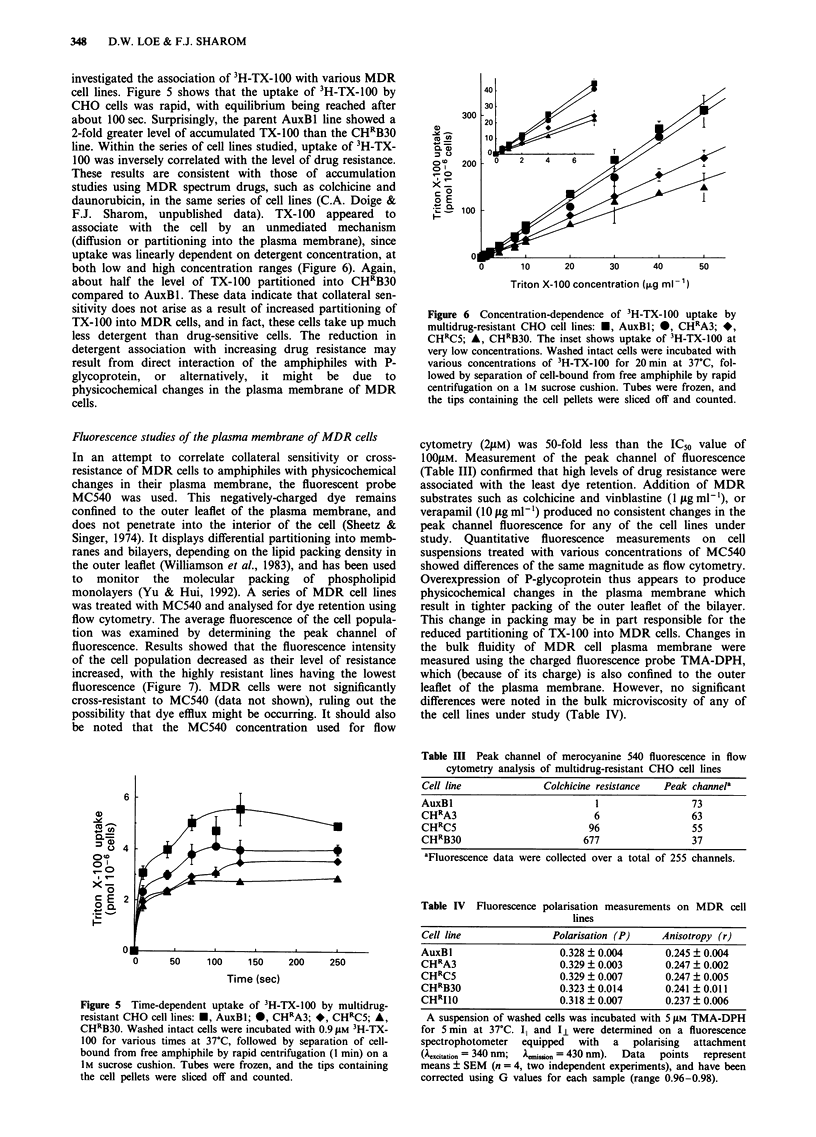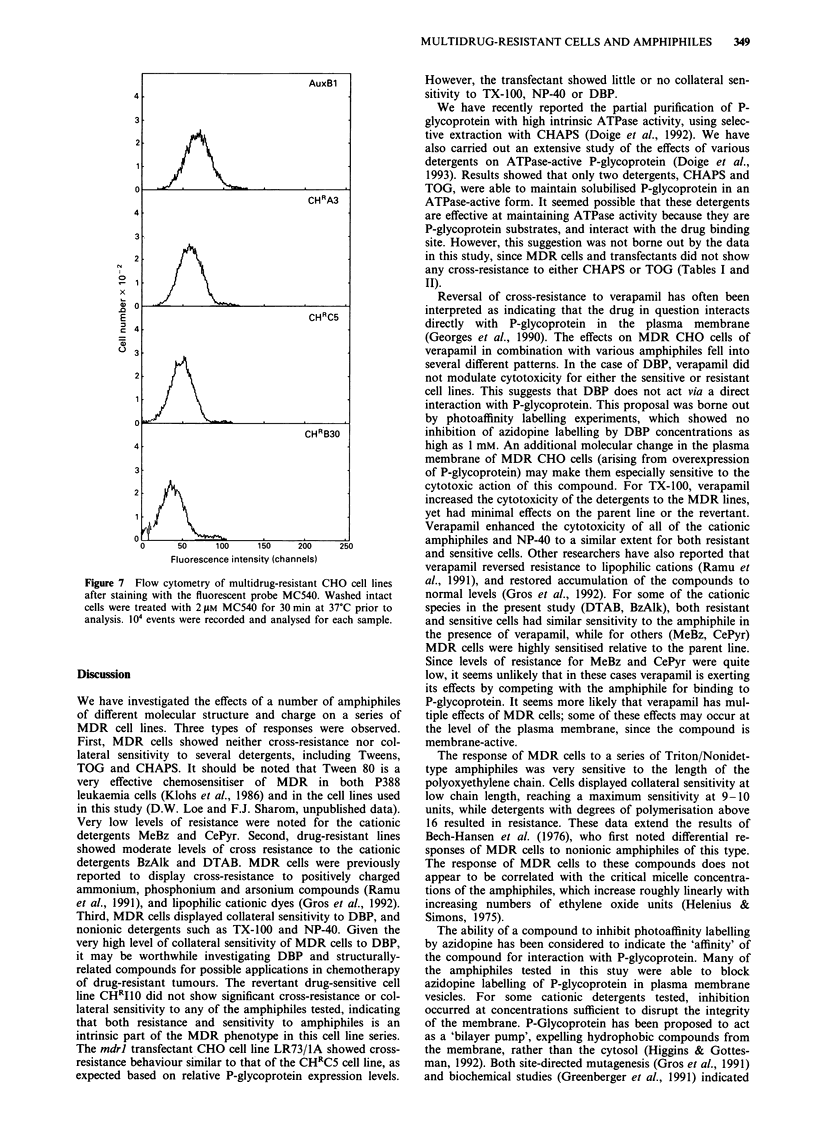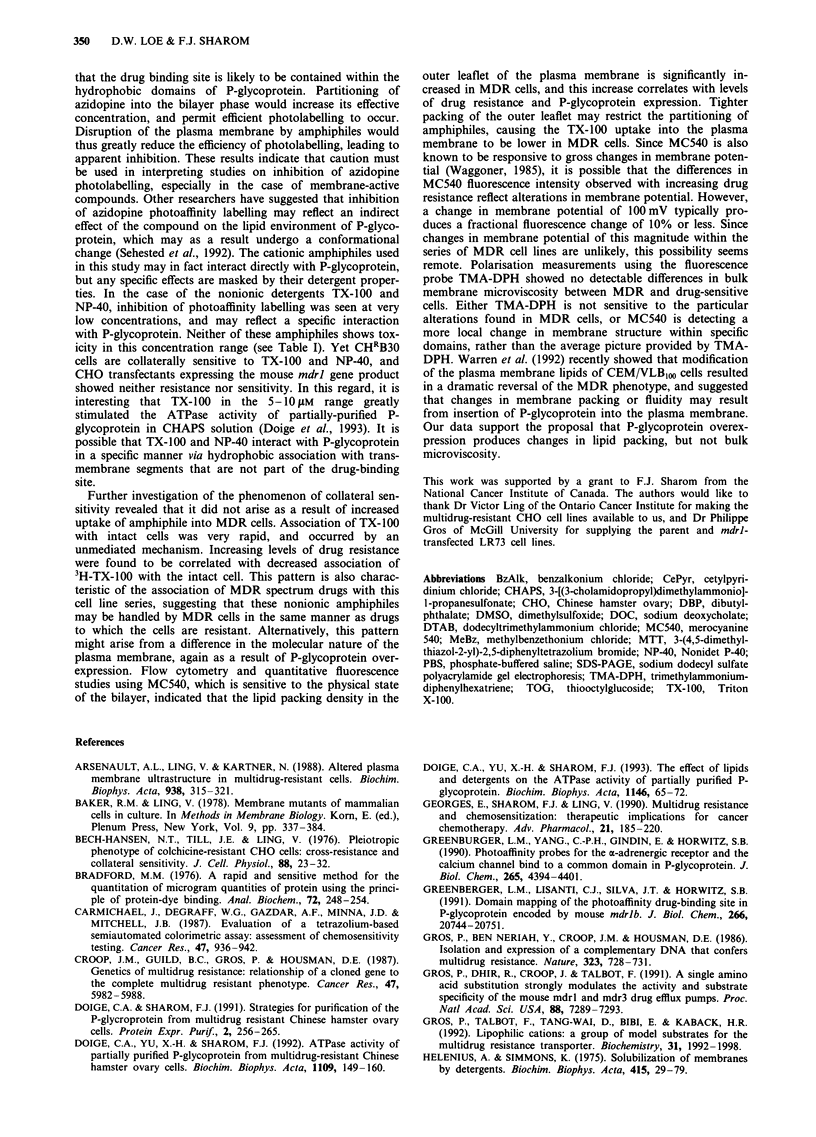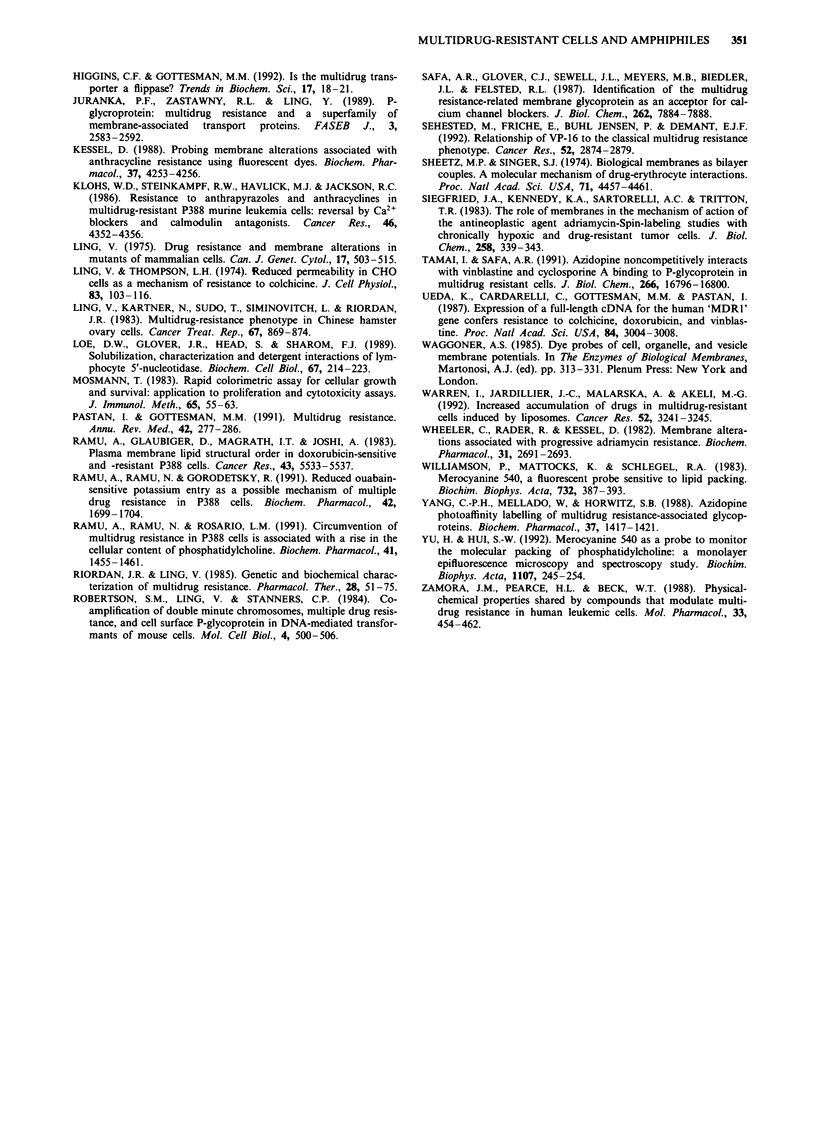# Interaction of multidrug-resistant Chinese hamster ovary cells with amphiphiles.

**DOI:** 10.1038/bjc.1993.338

**Published:** 1993-08

**Authors:** D. W. Loe, F. J. Sharom

**Affiliations:** Department of Chemistry and Biochemistry, University of Guelph, Ontario, Canada.

## Abstract

**Images:**


Br. J. Cancer (1993), 68, 342–351 © Macmillan Press Ltd., 1993

### Interaction of multidrug-resistant Chinese hamster ovary cells with amphiphiles

D.W. Loe & F.J. Sharom

*Guelph-Waterloo Centre for Graduate Work in Chemistry, Department of Chemistry and Biochemistry, University of Guelph, Ontario, N1G 2W1 Canada.*

**Summary** The interaction of membrane-active amphiphiles with a series of MDR Chinese hamster ovary (CHO) cell lines was investigated. Cross-resistance to cationic amphiphiles was observed, which was effectively sensitised by verapamil. MDR cells showed collateral sensitivity to polyoxyethylene amphiphiles (Triton X-100/Nonidet P-40), which reached a maximum at 9–10 ethylene oxide units. Resistant lines were also highly collaterally sensitive (17-fold) to dibutylphthalate. *mdrl* transfectants showed cross-resistance to cationic amphiphiles, but no collateral sensitivity to nonionic species. Triton X-100/Nonidet P-40 inhibited $^{3}$H-azidopine photoaffinity labelling at low concentrations, perhaps reflecting a specific interaction with P-glycoprotein.

Further investigation of the molecular basis of collateral sensitivity revealed that association of $^{3}$H-Triton X-100 with MDR cells reached steady state levels rapidly, and occurred by a non-mediated mechanism. The equilibrium level of X-100 uptake was inversely related to drug resistance. Collateral sensitivity is thus not a result of decreased Triton X-100 association with the cell.

The fluorescent probe merocyanine 540 was used to examine the MDR plasma membrane microenvironment for physicochemical changes. Increasing levels of drug resistance correlated with a progressive shift in the mean cell fluorescence to lower levels, which suggests that the packing density in the outer leaflet of MDR cells is increased relative to that of the drug-sensitive parent.

Multidrug resistance is a major barrier to the successful chemotherapeutic treatment of many human cancers. One common mechanism of resistance to drugs such as anthracyclines, the *Vinca* alkaloids, and epipodophyllotoxins, involves the overexpression of a 170–180 kDa integral membrane protein, known as the P-glycoprotein. This protein is believed to confer resistance by acting as an ATP-driven efflux pump, exporting drugs from the cell, and thus lowering their effective intracellular concentration (for reviews, see Juranka *et al.*, 1989; Georges *et al.*, 1990; Pastan & Gottesman, 1991). Transfection of the cDNA for P-glycoprotein (class I and II *mdr* genes) into a drug-sensitive cell is sufficient to confer the complete MDR phenotype (Gros *et al.*, 1986; Ueda *et al.*, 1987; Croop *et al.*, 1987), providing compelling evidence that this protein is a causative agent of drug resistance.

The model of P-glycoprotein as an ATP-driven efflux pump agrees well with existing data on drug accumulation and efflux in MDR cells. However, it does not readily account for the other pleiotropic changes seen in MDR cell membranes. For example, MDR is associated with increased sensitivity (collateral sensitivity) to membrane active agents, such as non-ionic detergents, local anaesthetics and steroids (Bech-Hansen *et al.*, 1976; Riordan & Ling, 1985). Changes in membrane permeability (Riordan & Ling, 1985), composition (Ramu *et al.*, 1991), ultrastructure (Arsenault *et al.*, 1988), and the physical properties of the membrane bilayer (Wheeler *et al.*, 1982; Ramu *et al.*, 1983; Siegfried *et al.*, 1983, Kessel, 1988) have also been reported. All of these observations suggest that the overexpression of P-glycoprotein leads to perturbations in membrane structure and function, perhaps arising in part from the insertion of a large hydrophobic protein into the plasma membrane. These secondary changes are important in determining the overall phenotype and responses of MDR cells, but their relationship to P-glycoprotein overexpression is not currently understood. Delineating the pleiotropic changes in the MDR cell membrane, and understanding their molecular basis, may be important in designing strategies for overcoming MDR clinically. For example, it may be possible to exploit the collateral sensitivity of MDR cells to eliminate cells with this phenotype from certain tumours.

The study of MDR has been greatly advanced by the use of continuous cell lines selected for resistance to one or more drugs in the MDR spectrum. Of the *in vitro* cell models that have been developed, one of the most useful has been Chinese hamster ovary (CHO) cells selected for resistance to colchicine by Ling and co-workers (Ling & Thompson, 1974). A series of these MDR cell lines derived from a common drug-sensitive parent is available (Bech-Hansen *et al.*, 1976; Ling, 1975), with increasing levels of colchicine resistance relative to the patent AuxB1 line, and increasing P-glycoprotein expression levels (Doige & Sharom, 1991). Transfected cells expressing specific *mdr* gene products have also proved to be useful in defining those cellular changes that arise as a result of the presence of P-glycoprotein in the plasma membrane. In this study, we have investigated the interaction of a series of drug-selected MDR CHO cell lines, and specific mouse *mdrl* CHO cell transfectants, with membrane-active amphiphiles, including several classes of detergents, in an effort to both delineate and understand the secondary changes occurring at the plasma membrane level in multidrug resistance.

### Materials and methods

Protein was determined by either the Pierce BCA assay system (Pierce, Rockford, IL) or the method of Bradford (Bradford, 1976) adapted for 96-well microtitre plates, using bovine serum albumin (crystallised and lyophilised; Sigma Chemical Co., St. Louis, MO) as a standard.

### *Growth of multidrug-resistant cell lines*

The AuxB1 parent CHO cell line (drug-sensitive), and the colchicine-selected lines CH$^{R}$A3 (6-fold resistant), CH$^{R}$C5 (96-fold resistant), CH$^{R}$B30 (680-fold resistant) have been described previously (Ling & Thompson, 1974; Ling, 1975; Robertson *et al.*, 1984; see also Table I). In addition, the revertant cell line CH$^{R}$I10 was selected from CH$^{R}$C5 (Baker & Ling, 1978), on the basis of the collateral sensitivity of the MDR line to 1-dehydrotestosterone. The I10 cell line showed a cytotoxic response to colchicine similar to that of the parent sensitive cell line (see Table I). All cell lines were

Correspondence: F.J. Sharom, Department of Chemistry and Biochemistry, University of Guelph, Guelph, Ontario, Canada N1G 2W1.

Received 4 August 1992; and in revised form 30 November 1992.

**Table I** Resistance and sensitivity of multidrug-resistant CHO cell lines to amphiphiles

| | $IC_{50}$ (μM)[a] | | | | |
|---|---|---|---|---|---|
| *Compound* | *AuxB1* | *CH[R]A3* | *CH[R]C5* | *CH[R]B30* | *CH[R]I10* |
| Colchicine | 0.13 | 0.75 (6)[b] | 12.5 (96) | 88.0 (680) | 0.25 (1.9) |
| + VRP | <0.03 (>4)[c] | 0.03 (25) | 0.5 (25) | 0.5 (180) | 0.03 (8.3) |
| VRP | 88.1 | 110.1 (1.3) | 143.2 (1.6) | 55.1 (0.6) | 48.5 (0.5) |
| *Cationic* | | | | | |
| BzAlk | 5.9 | 6.6 (1.1) | 29.5 (5.0) | 23.6 (4.0) | 5.9 (1.0) |
| + VRP | 2.8 (2.1) | 4.4 (1.5) | 2.9 (10) | 2.9 (8.1) | 3.7 (1.6) |
| MeBz | 39.0 | 36.8 (0.9) | 98. (2.5) | 98. (2.5) | 36.8 (0.9) |
| + VRP | 3.7 (11) | 3.7 (10) | 1.7 (58) | 3.2 (31) | 3.7 (10) |
| CePyr | 2.8 | 3.5 (1.3) | 5.6 (2.0) | 5.6 (2.0) | 1.4 (0.5) |
| + VRP | 1.4 (2.0) | 2.2 (1.6) | 0.4 (14) | 0.3 (19) | 0.8 (1.8) |
| DTAB | 13.0 | 16.2 (1.3) | 48.7 (3.7) | 48.7 (3.7) | 16.2 (1.2) |
| + VRP | 9.7 (1.3) | 9.7 (1.7) | 8.1 (6.0) | 8.8 (5.5) | 8.1 (2.0) |
| *Nonionic* | | | | | |
| TX-100 | 38.6 | 46.4 (1.2) | 23.2 (0.6) | 15.5 (0.4) | 41.7 (0.9) |
| + VRP | 38.6 (1.0) | 38.6 (1.2) | 10.8 (2.1) | 7.7 (2.0) | 54.1 (0.8) |
| NP-40 | 48.6 | 72.9 (1.5) | 24.3 (0.5) | 19.4 (0.4) | 48.6 (1.0) |
| + VRP | 16.2 (3.0) | 35.6 (2.0) | 12.2 (2.0) | 11.3 (1.7) | 51.9 (0.9) |
| Tween 80 | 1000 | 770 (0.8) | 1300 (1.3) | 923 (0.9) | 615 (0.6) |
| Tween 20 | 833 | 750 (0.9) | 1000 (1.2) | 833 (1.0) | 750 (0.9) |
| TOG | 325 | 324 (1.0) | 324 (1.0) | 324 (1.0) | 324 (1.0) |
| *Zwitterionic and anionic* | | | | | |
| CHAPS | 2400 | 2400 (1.0) | 2400 (1.0) | 2400 (1.0) | |
| DOC | 60.4 | 121 (2.0) | 169 (2.8) | 169 (2.8) | |
| *Other agents* | | | | | |
| DBP | 650 | 1080 (1.7) | 140 (0.2) | 40 (0.06) | 720 (1.0) |
| + VRP | 650 (1.0) | 1300 (0.8) | 140 (1.0) | 130 (0.3) | 720 (1.0) |

[a]$IC_{50}$ values were interpolated from cytotoxicity curves generated for each compound over a wide range of concentrations; [b]Numbers given in parentheses for each compound tested individually indicate the level of cross-resistance shown by that cell line, relative to the AuxB1 drug-sensitive cell line; [c]Numbers given in parentheses for each compound tested in combination with verapamil (VRP) indicate the chemosensitisation index, i.e. the ratio of the $IC_{50}$ value in the presence of verapamil to that in its absence.

maintained at 37°C in a humidified atmosphere of 5% $CO_2$ in α-MEM (Gibco Canada, Burlington, Ont.) supplemented with 10% bovine calf serum (defined/supplemented, heat-inactivated, Hyclone Laboratories, Logan, UT), penicillin (1000 U $ml^{-1}$), streptomycin (1 mg $ml^{-1}$) and 2 mM L-glutamine (all from Gibco). The CH[R]B30 cell line was maintained in 30 μg $ml^{-1}$ colchicine. Cell cultures were shown to be free of mycoplasma as tested with the Hoechst staining kit (Gibco).

Chinese hamster ovary LR73 cells, and murine *mdr1*-transfected LR73/1A cells were a gift of Dr Philippe Gros and were derived as described previously (Gros *et al.*, 1986). LR73/1A transfectants were grown in medium containing 0.1 μg $ml^{-1}$ doxorubicin. A comparison of the *mdr1* expression levels using Western blotting with the P-glycoprotein-specific monoclonal antibody C219 indicated that P-glycoprotein levels in the LR73/1A line were slightly lower than those of the CH[R]C5 line (X. Yu and F.J. Sharom, unpublished).

### *Cytotoxicity of amphiphiles towards MDR cell lines*

1-n-Octyl-β-D-thioglucopyranoside (thiooctylglucoside, TOG) was synthesised as described previously (Loe *et al.*, 1989). Verapamil and all other amphiphiles were purchased from Sigma. Trade names of compounds in the polyoxyethylene series (where *n* is the number of ethylene oxide units) are as follows: Triton X-15 ($n = 1$), 15-S-3 ($n = 3$), Nonidet P-14 ($n = 4$), Triton X-45 ($n = 5$), 15-S-7 and Nonidet P-7 ($n = 7$), Triton X-114 ($n = 8$), Nonidet P-40 (NP-40) ($n = 9$), Triton X-100 (TX-100) and X-100-R ($n = 9.6$), Triton X-102 ($n = 12-13$), 15-S-15 ($n = 15$), Triton X-165 ($n = 16$), Triton X-207 ($n = 20$), Triton X-305 ($n = 30$), Triton X-405 and X-405R ($n = 40$). Compounds in the 15-S series have a linear $C_{11}-C_{15}$ alkyl side chain, with no phenyl ring. Triton derivatives with the suffix R have a reduced cyclohexane ring in place of a phenyl ring. All compound were prepared as 20 mg $ml^{-1}$ stock solutions in dimethylsulfoxide (DMSO) or purified water, and diluted to 200 μg $ml^{-1}$ in α-MEM. The final DMSO concentration (1% v/v) had no effect on the growth rate or viability of any of the cell lines.

Cytotoxicity of various amphiphiles to MDR cell lines was determined by a modification of the MTT dye reduction assay described previously (Mosmann, 1983; Carmichael *et al.*, 1987). Briefly, monolayer cell cultures were harvested by trypsinisation, followed by centrifugation at 1000 *g* for 10 min and resuspension in a suitable volume of α-MEM (cell concentration around $10^4$ $ml^{-1}$). Amphipile solutions were prepared as serial dilutions and dispensed as 100 μl aliquots into triplicate wells of a 96-well culture plate (Nunc). Plates were incubated as 37°C for 96 h, after which time cell growth was assayed as outlined below.

3-(4,5-Dimethylthiazol-2-yl)-2,5-diphenyltetrazolium bromide (MTT) was prepared as a 5 mg $ml^{-1}$ solution in phosphate-buffered saline (PBS; 50 mM phosphate in 0.15 M NaCl) and sterilised using a 0.22 μm filter prior to use. MTT stock solution was diluted to 1 mg $ml^{-1}$ with α-MEM and 50 μl was added to each well (50 μg MTT per well). After incubation for 4 h at 37°C, the culture medium in each well was aspirated off using a multichannel pipettor and 150 μl DMSO was added to solubilise intracellular formazan crystals. Absorbance was measured using a Titertek ELISA plate reader with a 580 nm filter (absorbance at 580 nm was 80% of that at the $\lambda_{max}$ of 514 nm). Absorbance values were corrected for MTT reduction measured in the absence of cells. Relative cell growth was calculated by comparison to growth in the absence of amphiphile, and the data were plotted as relative cell growth (% control) *vs* log drug concentration. $IC_{50}$ values were determined by interpolation from cytotoxicity plots. Cross-resistance of each cell line towards each compound tested was calculated as the ratio of the $IC_{50}$ value for the cell line relative to the AuxB1 drug-sensitive parent line. Values greater than 1.0 indicate cross-resistance, while values less than 1.0 indicate collateral sensitivity. To test the effect of verapamil on amphiphile toxicity, cell lines were grown as above in the presence of various concentrations of amphiphiles, together with 20μM verapamil. The chemosensitisation index for verapamil tested in combination with each compound was calculated as the ratio of the $IC_{50}$ value in the presence of verapamil to that in its absence. Values greater than 1.0 indicate that verapamil sensitised the cell line to the cytotoxicity of that compound.

### *Photoaffinity labelling*

Plasma membrane vesicles were prepared from $CH^RC5$ cells by nitrogen cavitation, followed by discontinuous sucrose gradient sedimentation, as described previously (Doige & Sharom, 1991). Vesicles were resuspended in buffer (10 mM Tris, 0.25 M sucrose, pH 7.4) containing 1 $\mu g\ ml^{-1}$ aprotinin, leupeptin, and pepstatin A, and 25 $\mu g\ ml^{-1}$ phenylmethylsulfonyl fluoride, (all from Sigma). Photoaffinity labelling was carried out according to Safa *et al.* (1987). Briefly, membrane vesicles (20 $\mu g$ of protein) were incubated for 1 h in the dark with 200 nM $^3H$-azidopine (52 Ci $mmol^{-1}$; Amersham Canada, Oakville, Ont.), in the presence of increasing concentrations of various amphiphiles. Samples were exposed to a 30 W ultraviolet lamp at a distance of 8 cm for 20 min, and then analysed by sodium dodecyl sulfate polyacrylamide gel electrophoresis (SDS-PAGE), followed by fluorography.

### *Association of Triton X-100 with MDR cells*

Cells were grown in 175 $cm^2$ culture flasks for 4 days, harvested by trypsinisation and centrifugation at 1000 *g* for 10 min, resuspended in α-MEM/10% serum, and counted using a Coulter counter. Cell suspensions were agitated at 37°C in a gyrorotatory shaker for 2 h, sedimented, and resuspended in medium at a concentration of $4 \times 10^6$ cells $ml^{-1}$. For determination of time-dependent uptake, 200 $\mu l$ of 1.8$\mu$M Triton X-100 (TX-100) containing 0.4 $\mu Ci$ $^3H$-TX-100 (2 Ci $mmol^{-1}$; DuPont Canada, Mississauga, Ont.) was added to a 200 $\mu l$ aliquot of cell suspension in 1 ml round-bottomed polypropylene tubes, to give a final TX-100 concentration of 0.9$\mu$M. After shaking for various times at 37°C, aliquots were removed, layered over ice-cold 1 M sucrose/PBS, and centrifuged at 15,000 *g* for 1 min to separate cells from unbound detergent. Tubes were frozen in liquid nitrogen and the tips containing the cell pellet were sliced off. After vigorous agitation of the tips in Ready-Safe scintillant (Beckman Canada, Mississauga, Ont.) using a vortexer, the samples were counted for $^3H$ by liquid scintillation counting. For concentration-dependent uptake, TX-100 solutions were prepared by serial dilution of a 150$\mu$M stock solution containing 1 $\mu Ci\ ml^{-1}$ $^3H$-TX-100, and cell-associated detergent was measured as above.

### *Flow cytometry*

Trypsinised cells were diluted to $10^5$ cells $ml^{-1}$ in α-MEM/10% serum and agitated for 30 min at 37°C in 2$\mu$M merocyanine 540 (MC540, Sigma), in either the absence or presence of 1$\mu$M colchicine or vinblastine. Analysis was carried out using an EPICS V Flow Cytometer-Cell Sorter (Coulter Electronics, Hialeach, FL), equipped with a 5W argon-ion laser (Coherent, Palo Alto, CA), a multiple data acquisition and display system (MDADS) and a Coulter Electronics Autoclone. Forward-angle light scatter (FALS) was used to gate CHO cells. Cells were analysed using a 500 W laser at 488 nm, and the log of integrated red fluorescence (LIRFL) was determined at 560 nm. Frequency histograms for each cell line were plotted as the number of events *vs* fluorescence intensity (channel number). $10^4$ events (cells) were recorded and analysed for each sample. All cell lines were labelled with MC540 and analysed within the same experiment, to generate an internally consistent set of data.

### *Fluorescence anisotropy of TMA-DPH in MDR cell membranes*

Trypsinised cells were suspended in buffer at a concentration of $1 \times 10^6$ cells $ml^{-1}$, and allowed to stand at 37°C for 15 min. An aliquot of the suspension (3 ml) was dispensed into a cuvette and mixed with 3 $\mu l$ of 5 mM trimethylammonium-diphenylhexatriene (TMA-DPH, Molecular Probes, Eugene, OR) in dimethylformamide, $I_{\parallel}$ and $I_{\perp}$ were determined on a Hitachi F-2000 fluorescence spectrophotometer equipped with a polarising attachment ($\lambda_{excitation} = 340$ nm; $\lambda_{emission} = 430$ nm). Fluorescence polarisation (P) and anisotropy (r) were calculated according to the following equations:

$$P = (I_{\parallel} - I_{\perp})/(I_{\parallel} + I_{\perp})$$

$$r = (I_{\parallel} - I_{\perp})/(I_{\parallel} + 2I_{\perp})$$

G values were routinely measured for each sample (range 0.96–0.98), and the data were corrected accordingly.

### Results

### *Cross-resistance and collateral sensitivity of MDR cells to amphiphiles and effects of verapamil*

A series of MDR cell lines with different degrees of colchicine resistance and different P-glycoprotein expression levels was tested for cross-resistance or collateral sensitivity to a variety of amphiphiles of differing molecular structure and charge (see Figure 1). The ability of verapamil, a well-known chemosensitiser, to modify resistance or sensitivity was also determined. Table I indicates the cross-resistance or collateral sensitivity of the cell lines to various amphiphiles, as well as the chemosensitisation index, which is a measure of the ability of verapamil to sensitise the cell line to the cytotoxic effects of each compound. Many compounds that fall within the MDR spectrum are cationic (Zamora *et al.*, 1988), so it was of interest to assess the interaction of drug-resistant lines with positively-charged amphiphiles. MDR cells demonstrated similar patterns of resistance to the cationic detergents benzalkonium chloride (BzAlk), methylbenzethonium chloride (MeBz), cetylpyridinium chloride (CePyr), and dodecyltrimethylammonium chloride (DTAB) (Table I). Figure 2 shows a resistance profile for BzAlk, which is typical of this class of amphiphiles. MDR cell lines with high P-glycoprotein expression levels showed moderate resistance to BzAlk and DTAB (4- to 5-fold, see Table I), and lower cross-resistance to MeBz and CePyr. The drug-sensitive revertant line $CH^RI10$ displayed resistance characteristics very similar to that of AuxB1, confirming that resistance to cationic detergents is related to the MDR phenotype. Verapamil had the effect of moving the amphiphile $IC_{50}$ for both the resistant and sensitive cell lines to much lower values (Figure 2). Although this effect was seen for all four cationic detergents, it was most pronounced for MeBz, where the chemosensitisation index reached 58 for $CH^RC5$. The MDR lines also showed substantially lower $IC_{50}$ values for CePyr than the sensitive parent in the presence of verapamil. Verapamil showed similar cytotoxicity towards all of the cell lines, and the concentration used for chemosensitisation (20$\mu$M) was well below toxic levels. CHO cells appear to very sensitive to the cytotoxic actions of cationic detergents, with $IC_{50}$ values in the range 3–40$\mu$M for AuxB1 and 4–65$\mu$M for $CH^RC5$.

MDR cells have been reported to show collateral sensitivity towards certain nonionic detergents (Bech-Hansen *et al.*, 1976; Riordan & Ling, 1985). As shown in Table I and Figure 2, MDR cell lines exhibited significant collateral sensitivity towards the polyoxyethylene ether amphiphiles TX-100 and NP-40, with the highly resistant line $CH^RB30$ being several-fold more sensitive to their cytotoxic action than the parent line. Again, the revertant line did not display collateral sensitivity to these detergents. The addition of verapamil did not affect the sensitivity of AuxB1 to TX-100, but shifted the $IC_{50}$ value for $CH^RC5$ and $CH^RB30$ about 2-fold lower (Figure 2). In the case of NP-40, verapamil increased the cytotoxicity of the detergent for both sensitive and resistant lines between 1.7- and 3-fold (Table I and Figure 2). Cells were moderately sensitive to the cytotoxic

**Figure 1** Structure of some amphiphiles used in this study. **a**, BzAlk ($n = 12$, with some 14 and 16); **b**, MeBz; **c**, CePyr; **d**, DTAB; **e**, Triton-Nonidet series ($n = 1$–$40$); **f**, TOG; **g**, DOC; **h**, DBP; **i**, CHAPS; **j**, Tween series ($w + x + y + z = 20$; $n = 11$ for Tween 20, and $n = 17$ unsaturated for Tween 80).

**Figure 2** Effect of amphiphiles on the growth *in vitro* of the CHO cell lines AuxB1 (drug-sensitive parent; ■, □), and $CH^{R}C5$ (multidrug-resistant; ◆, ◇). The closed symbols indicate experiments conducted in the presence of drug only, while the open symbols indicate experiments conducted in the presence of drug and 20 μM verapamil. Limits of error ($n = 3$) are included within the size of the symbols.

action of TX-100 and NP-40 ($IC_{50}$ values in the range 15–73μM). In contrast, drug-resistant cell lines showed no collateral sensitivity or cross-resistance to other nonionic detergents such as Tweens 20 and 80 (polyoxyethylene sorbitans) and TOG (Table I). Detergent concentrations approaching the mM range were necessary to cause cell killing.

MDR cells showed a remarkably high level of collateral sensitivity to the compound dibutylphthalate (DBP) (Figure 2). $CH^RB30$ was 17-fold more sensitive to DBP cytotoxicity than the drug-sensitive parent, with an $IC_{50}$ value of 40μM (Table I). The addition of verapamil had little effect on DBP cytotoxicity. The revertant cell line was not collaterally sensitive to DBP. The molecular basis of such high levels of collateral sensitivity is currently unknown.

MDR cells showed no cross-resistance to the zwitterionic detergent 3-[(3-cholamido-propyl)dimethylammonio]-1-propanesulfonate (CHAPS) (Table I), and were remarkably resistant to killing by this compound, showing $IC_{50}$ values of 2.4 mM. Thus CHO cells can tolerate very high levels of agents that are membrane solubilisers. Low levels of cross-resistance were also observed to sodium deoxycholate (DOC), an anionic detergent, which was moderately cytotoxic (Table I).

CHO cell lines transfected with the mouse *mdr1* gene were also examined for cross-resistance and collateral sensitivity toward nonionic detergents and DBP. Transfectants were highly cross-resistant to drugs within the MDR spectrum (Gros *et al.*, 1986, and Table II). As shown in Table II, they showed a small degree of cross-resistance to the cationic amphiphiles BzAlk, MeBz, CePyr and DTAB. As with the colchicine-selected MDR cell lines, verapamil greatly increased the toxicity of these compounds to both parent and transfectant lines, which showed similar $IC_{50}$ values in the presence of the chemosensitiser. *mdr1* transfectants showed little or no collateral sensitivity to TX-100, NP-40, and DBP. This suggests that the extent of collateral sensitivity is dependent on the particular parent cell line.

**Table II** Resistance and sensitivity of *mdr1* transfectant CHO cell lines to amphiphiles

| *Compound* | *$IC_{50}$ (μM)*[a] *LR73* | *LR73/1A*[b] |
|---|---|---|
| Colchicine | 0.045 | 1.26 (28)[c] |
| + VRP | 0.014 (3.2)[d] | 0.04 (33) |
| *Cationic* | | |
| BzAlk | 22.0 | 44.0 (2.0) |
| + VRP | 2.66 (8.3) | 2.95 (15) |
| MeBz | 36.7 | 75.6 (2.1) |
| + VRP | 5.83 (6.3) | 17.3 (4.4) |
| CePyr | 6.98 | 16.7 (2.4) |
| + VRP | 1.53 (4.6) | 1.53 (11) |
| DTAB | 39.0 | 94.3 (2.4) |
| + VRP | 10.4 (3.8) | 5.85 (16) |
| *Nonionic* | | |
| TX-100 | 46.4 | 49.5 (1.1) |
| + VRP | 29.4 (1.6) | 35.5 (1.4) |
| NP-40 | 58.0 | 49.8 (0.86) |
| Tween 80 | 928 | 928 (1.0) |
| Tween 20 | 701 | 825 (1.2) |
| TOG | 362 | 458 (1.3) |
| *Zwitterionic and anionic* | | |
| CHAPS | 4075 | 5216 (1.3) |
| DOC | 362 | 458 (1.3) |
| *Other agents* | | |
| DBP | 151 | 151 (1.0) |

[a]$IC_{50}$ values were interpolated from cytotoxicity curves generated for each compound over a wide range of concentrations; [b] Transfectants expressing the mouse *mdr1* gene; [c]Numbers given in parentheses for each compound tested individually indicate the level of cross-resistance shown by that cell line, relative to the AuxB1 drug-sensitive cell line; [d] Numbers given in parentheses for each compound tested in combination with verapamil (VRP) indicate the chemosensitisation index; i.e. the ratio of the $IC_{50}$ value in the presence of verapamil to that in its absence.

The MDR cell line $CH^RPha^R$, which was derived from $CH^RC5$ by selection with the toxic lectin phytohemagglutinin (Ling *et al.*, 1983), has greatly reduced N-linked glycosylation of membrane proteins, resulting in expression of a severely underglycosylated P-glycoprotein of molecular mass 140 kDa (D.W. Loe and F.J. Sharom, unpublished). This cell line behaved very similarly to the $CH^RC5$ parent with respect to cross-resistance and collateral sensitivity to the compounds tested in this study (data not shown), indicating that the extent of P-glycoprotein glycosylation does not play a role in either resistance or sensitivity to amphiphiles.

*Effect of detergent structure on collateral sensitivity*

We further investigated the phenomenon of collateral sensitivity by determining the effect on MDR cells of a series of polyoxyethylene detergents with similar overall chemical structures, but different molecular size. The $IC_{50}$ for these amphiphiles decreased over 10-fold as the number of ethylene oxide units (*n*) approached 10, and then gradually increased back to its former value as *n* reached 30 (Figure 3a). Species with $n = 9-10$ were considerably more toxic to the $CH^RB30$ cells than the drug-sensitive parent. As shown in Figure 3b, the degree of collateral sensitivity of MDR cells to these detergents increased with *n*, reaching a maximum at $n = 9-10$ with the compounds TX-100 and NP-40). At higher values of *n*, the cells displayed cross-resistance. $CH^RB30$ cells were considerably more sensitive to detergent analogues with reduced cyclohexane rings, rather than phenyl rings, but the same *n* value.

**Figure 3** **a**, Toxicity of the multidrug-resistant cell line $CH^RB30$, and the drug-sensitive parent AuxB1 to nonionic polyoxyethylene detergents containing increasing numbers of ethylene oxide units. **b**, Relative resistance and collateral sensitivity of $CH^RB30$ to the detergents. Values of resistance below 1.0 are indicative of collateral sensitivity. Open symbols indicate detergent analogues with a reduced cyclohexane ring, rather than a phenyl ring. A full list of the trade names of the detergents used is given in Materials and methods.

### *Ability of amphiphiles to block azidopine photoaffinity labelling of P-glycoprotein*

Azidopine has been shown to specifically photoaffinity label P-glycoprotein in the plasma membrane of MDR cells (Safa *et al.*, 1987; Yang *et al.*, 1988). The labelling can be prevented by increasing concentrations of other compounds known to be substrates for the multidrug transporter (e.g. vinblastine, verapamil). It was suggested that azidopine modifies drug binding to P-glycoprotein by interacting with the protein directly at either the drug-binding site itself, or an allosteric site (Tamai & Safa, 1991). Since MDR cells display cross-resistance to cationic detergents that can be reversed by verapamil, these compounds may also interact directly with P-glycoprotein. It was therefore of interest to determine whether any of the amphiphiles for which cross-resistance or collateral sensitivity was identified in this study, are able to compete with azidopine for photolabelling of P-glycoprotein. As shown in Figure 4, $^3$H-azidopine photolabelled a 170–180 kDa protein in the CH$^R$C5 plasma membrane, corresponding to P-glycoprotein. Labelling was blocked by addition of increasing concentrations of vinblastine, with 50% inhibition observed at a 25- to 50-fold excess of vinblastine, in the range 5–10µM (Figure 4a). These data are in agreement with previous reports (Greenberger *et al.*, 1990). TX-100 and NP-40 also inhibited azidopine labelling at very low concentrations, with an $IC_{50}$ in the range 4–8 µM (Figures 4b and 4c). By this criterion, these nonionic detergents would be considered high affinity P-glycoprotein substrates, yet other data reported in this work indicate that MDR cells and transfectants are clearly not cross-resistant to these compounds. DBP did not affect photoaffinity labelling at concentrations as high as 1 mM (Figure 4d), indicating that the ability to inhibit photolabelling is not linked to collateral sensitivity. Addition of the cationic amphiphiles BzAlk (Figure 4e) and MeBz (Figure 4f) produced inhibition of photolabelling ($IC_{50}$ values of 250 and 500µM, respectively), but only at concentrations which are probably large enough to produce some membrane disruption. It seems likely that an intact membrane is necessary for photoaffinity labelling by azidopine to proceed efficiently. These amphiphiles probably inhibit labelling by disrupting the membrane, rather than competing directly with azidopine for the drug binding site on P-glycoprotein. This suggests that care should be taken in the interpretation of photoaffinity labelling inhibition data, especially if the compounds being tested are membrane-active.

### *Uptake of $^3$H-Triton X-100 by MDR cells*

The enhanced sensitivity of MDR cells to TX-100 could arise from an increase in the levels of detergent that partition into the plasma membrane. In an attempt to determine whether this was the molecular basis for collateral sensitivity, we

**Figure 4** Effect of drugs and amphiphiles on azidopine photoaffinity labelling of P-glycoprotein in CH$^R$C5 plasma membrane vesicles. **a**, vinblastine; **b**, TX-100; **c**, NP-40; **d**, DBP; **e**, BzAlk; **f**, MeBz. Concentrations of the various amphiphiles are shown at the bottom of the gel. Arrows and numbers to the left of the gels indicate the position of molecular mass markers in kDa.

investigated the association of $^3$H-TX-100 with various MDR cell lines. Figure 5 shows that the uptake of $^3$H-TX-100 by CHO cells was rapid, with equilibrium being reached after about 100 sec. Surprisingly, the parent AuxB1 line showed a 2-fold greater level of accumulated TX-100 than the CH$^R$B30 line. Within the series of cell lines studied, uptake of $^3$H-TX-100 was inversely correlated with the level of drug resistance. These results are consistent with those of accumulation studies using MDR spectrum drugs, such as colchicine and daunorubicin, in the same series of cell lines (C.A. Doige & F.J. Sharom, unpublished data). TX-100 appeared to associate with the cell by an unmediated mechanism (diffusion or partitioning into the plasma membrane), since uptake was linearly dependent on detergent concentration, at both low and high concentration ranges (Figure 6). Again, about half the level of TX-100 partitioned into CH$^R$B30 compared to AuxB1. These data indicate that collateral sensitivity does not arise as a result of increased partitioning of TX-100 into MDR cells, and in fact, these cells take up much less detergent than drug-sensitive cells. The reduction in detergent association with increasing drug resistance may result from direct interaction of the amphiphiles with P-glycoprotein, or alternatively, it might be due to physicochemical changes in the plasma membrane of MDR cells.

**Figure 6** Concentration-dependence of $^3$H-TX-100 uptake by multidrug-resistant CHO cell lines: ■, AuxB1; ●, CH$^R$A3; ◆, CH$^R$C5; ▲, CH$^R$B30. The inset shows uptake of $^3$H-TX-100 at very low concentrations. Washed intact cells were incubated with various concentrations of $^3$H-TX-100 for 20 min at 37°C, followed by separation of cell-bound from free amphiphile by rapid centrifugation on a 1 M sucrose cushion. Tubes were frozen, and the tips containing the cell pellets were sliced off and counted.

*Fluorescence studies of the plasma membrane of MDR cells*

In an attempt to correlate collateral sensitivity or cross-resistance of MDR cells to amphiphiles with physicochemical changes in their plasma membrane, the fluorescent probe MC540 was used. This negatively-charged dye remains confined to the outer leaflet of the plasma membrane, and does not penetrate into the interior of the cell (Sheetz & Singer, 1974). It displays differential partitioning into membranes and bilayers, depending on the lipid packing density in the outer leaflet (Williamson *et al.*, 1983), and has been used to monitor the molecular packing of phospholipid monolayers (Yu & Hui, 1992). A series of MDR cell lines was treated with MC540 and analysed for dye retention using flow cytometry. The average fluorescence of the cell population was examined by determining the peak channel of fluorescence. Results showed that the fluorescence intensity of the cell population decreased as their level of resistance increased, with the highly resistant lines having the lowest fluorescence (Figure 7). MDR cells were not significantly cross-resistant to MC540 (data not shown), ruling out the possibility that dye efflux might be occurring. It should also be noted that the MC540 concentration used for flow cytometry (2μM) was 50-fold less than the $IC_{50}$ value of 100μM. Measurement of the peak channel of fluorescence (Table III) confirmed that high levels of drug resistance were associated with the least dye retention. Addition of MDR substrates such as colchicine and vinblastine (1 μg ml$^{-1}$), or verapamil (10 μg ml$^{-1}$) produced no consistent changes in the peak channel fluorescence for any of the cell lines under study. Quantitative fluorescence measurements on cell suspensions treated with various concentrations of MC540 showed differences of the same magnitude as flow cytometry. Overexpression of P-glycoprotein thus appears to produce physicochemical changes in the plasma membrane which result in tighter packing of the outer leaflet of the bilayer. This change in packing may be in part responsible for the reduced partitioning of TX-100 into MDR cells. Changes in the bulk fluidity of MDR cell plasma membrane were measured using the charged fluorescence probe TMA-DPH, which (because of its charge) is also confined to the outer leaflet of the plasma membrane. However, no significant differences were noted in the bulk microviscosity of any of the cell lines under study (Table IV).

**Figure 5** Time-dependent uptake of $^3$H-TX-100 by multidrug-resistant CHO cell lines: ■, AuxB1; ●, CH$^R$A3; ◆, CH$^R$C5; ▲, CH$^R$B30. Washed intact cells were incubated with 0.9 μM $^3$H-TX-100 for various times at 37°C, followed by separation of cell-bound from free amphiphile by rapid centrifugation (1 min) on a 1 M sucrose cushion. Tubes were frozen, and the tips containing the cell pellets were sliced off and counted.

**Table III** Peak channel of merocyanine 540 fluorescence in flow cytometry analysis of multidrug-resistant CHO cell lines

| *Cell line* | *Colchicine resistance* | *Peak channel*[a] |
|---|---|---|
| AuxB1 | 1 | 73 |
| CH$^R$A3 | 6 | 63 |
| CH$^R$C5 | 96 | 55 |
| CH$^R$B30 | 677 | 37 |

[a]Fluorescence data were collected over a total of 255 channels.

**Table IV** Fluorescence polarisation measurements on MDR cell lines

| *Cell line* | *Polarisation (P)* | *Anisotropy (r)* |
|---|---|---|
| AuxB1 | 0.328 ± 0.004 | 0.245 ± 0.004 |
| CH$^R$A3 | 0.329 ± 0.003 | 0.247 ± 0.002 |
| CH$^R$C5 | 0.329 ± 0.007 | 0.247 ± 0.005 |
| CH$^R$B30 | 0.323 ± 0.014 | 0.241 ± 0.011 |
| CH$^R$I10 | 0.318 ± 0.007 | 0.237 ± 0.006 |

A suspension of washed cells was incubated with 5 μM TMA-DPH for 5 min at 37°C. $I_{\parallel}$ and $I_{\perp}$ were determined on a fluorescence spectrophotometer equipped with a polarising attachment ($\lambda_{excitation}$ = 340 nm; $\lambda_{emission}$ = 430 nm). Data points represent means ± SEM ($n$ = 4, two independent experiments), and have been corrected using G values for each sample (range 0.96–0.98).

**Figure 7** Flow cytometry of multidrug-resistant CHO cell lines after staining with the fluorescent probe MC540. Washed intact cells were treated with 2 μM MC540 for 30 min at 37°C prior to analysis. $10^4$ events were recorded and analysed for each sample.

### Discussion

We have investigated the effects of a number of amphiphiles of different molecular structure and charge on a series of MDR cell lines. Three types of responses were observed. First, MDR cells showed neither cross-resistance nor collateral sensitivity to several detergents, including Tweens, TOG and CHAPS. It should be noted that Tween 80 is a very effective chemosensitiser of MDR in both P388 leukaemia cells (Klohs *et al.*, 1986) and in the cell lines used in this study (D.W. Loe and F.J. Sharom, unpublished data). Very low levels of resistance were noted for the cationic detergents MeBz and CePyr. Second, drug-resistant lines showed moderate levels of cross resistance to the cationic detergents BzAlk and DTAB. MDR cells were previously reported to display cross-resistance to positively charged ammonium, phosphonium and arsonium compounds (Ramu *et al.*, 1991), and lipophilic cationic dyes (Gros *et al.*, 1992). Third, MDR cells displayed collateral sensitivity to DBP, and nonionic detergents such as TX-100 and NP-40. Given the very high level of collateral sensitivity of MDR cells to DBP, it may be worthwhile investigating DBP and structurally-related compounds for possible applications in chemotherapy of drug-resistant tumours. The revertant drug-sensitive cell line $CH^RI10$ did not show significant cross-resistance or collateral sensitivity to any of the amphiphiles tested, indicating that both resistance and sensitivity to amphiphiles is an intrinsic part of the MDR phenotype in this cell line series. The *mdrl* transfectant CHO cell line LR73/1A showed cross-resistance behaviour similar to that of the $CH^RC5$ cell line, as expected based on relative P-glycoprotein expression levels. However, the transfectant showed little or no collateral sensitivity to TX-100, NP-40 or DBP.

We have recently reported the partial purification of P-glycoprotein with high intrinsic ATPase activity, using selective extraction with CHAPS (Doige *et al.*, 1992). We have also carried out an extensive study of the effects of various detergents on ATPase-active P-glycoprotein (Doige *et al.*, 1993). Results showed that only two detergents, CHAPS and TOG, were able to maintain solubilised P-glycoprotein in an ATPase-active form. It seemed possible that these detergents are effective at maintaining ATPase activity because they are P-glycoprotein substrates, and interact with the drug binding site. However, this suggestion was not borne out by the data in this study, since MDR cells and transfectants did not show any cross-resistance to either CHAPS or TOG (Tables I and II).

Reversal of cross-resistance to verapamil has often been interpreted as indicating that the drug in question interacts directly with P-glycoprotein in the plasma membrane (Georges *et al.*, 1990). The effects on MDR CHO cells of verapamil in combination with various amphiphiles fell into several different patterns. In the case of DBP, verapamil did not modulate cytotoxicity for either the sensitive or resistant cell lines. This suggests that DBP does not act *via* a direct interaction with P-glycoprotein. This proposal was borne out by photoaffinity labelling experiments, which showed no inhibition of azidopine labelling by DBP concentrations as high as 1 mM. An additional molecular change in the plasma membrane of MDR CHO cells (arising from overexpression of P-glycoprotein) may make them especially sensitive to the cytotoxic action of this compound. For TX-100, verapamil increased the cytotoxicity of the detergents to the MDR lines, yet had minimal effects on the parent line or the revertant. Verapamil enhanced the cytotoxicity of all of the cationic amphiphiles and NP-40 to a similar extent for both resistant and sensitive cells. Other researchers have also reported that verapamil reversed resistance to lipophilic cations (Ramu *et al.*, 1991), and restored accumulation of the compounds to normal levels (Gros *et al.*, 1992). For some of the cationic species in the present study (DTAB, BzAlk), both resistant and sensitive cells had similar sensitivity to the amphiphile in the presence of verapamil, while for others (MeBz, CePyr) MDR cells were highly sensitised relative to the parent line. Since levels of resistance for MeBz and CePyr were quite low, it seems unlikely that in these cases verapamil is exerting its effects by competing with the amphiphile for binding to P-glycoprotein. It seems more likely that verapamil has multiple effects of MDR cells; some of these effects may occur at the level of the plasma membrane, since the compound is membrane-active.

The response of MDR cells to a series of Triton/Nonidet-type amphiphiles was very sensitive to the length of the polyoxyethylene chain. Cells displayed collateral sensitivity at low chain length, reaching a maximum sensitivity at 9–10 units, while detergents with degrees of polymerisation above 16 resulted in resistance. These data extend the results of Bech-Hansen *et al.* (1976), who first noted differential responses of MDR cells to nonionic amphiphiles of this type. The response of MDR cells to these compounds does not appear to be correlated with the critical micelle concentrations of the amphiphiles, which increase roughly linearly with increasing numbers of ethylene oxide units (Helenius & Simons, 1975).

The ability of a compound to inhibit photoaffinity labelling by azidopine has been considered to indicate the 'affinity' of the compound for interaction with P-glycoprotein. Many of the amphiphiles tested in this stuy were able to block azidopine labelling of P-glycoprotein in plasma membrane vesicles. For some cationic detergents tested, inhibition occurred at concentrations sufficient to disrupt the integrity of the membrane. P-Glycoprotein has been proposed to act as a 'bilayer pump', expelling hydrophobic compounds from the membrane, rather than the cytosol (Higgins & Gottesman, 1992). Both site-directed mutagenesis (Gros *et al.*, 1991) and biochemical studies (Greenberger *et al.*, 1991) indicated that the drug binding site is likely to be contained within the hydrophobic domains of P-glycoprotein. Partitioning of azidopine into the bilayer phase would increase its effective concentration, and permit efficient photolabelling to occur. Disruption of the plasma membrane by amphiphiles would thus greatly reduce the efficiency of photolabelling, leading to apparent inhibition. These results indicate that caution must be used in interpreting studies on inhibition of azidopine photolabelling, especially in the case of membrane-active compounds. Other researchers have suggested that inhibition of azidopine photoaffinity labelling may reflect an indirect effect of the compound on the lipid environment of P-glycoprotein, which may as a result undergo a conformational change (Sehested *et al.*, 1992). The cationic amphiphiles used in this study may in fact interact directly with P-glycoprotein, but any specific effects are masked by their detergent properties. In the case of the nonionic detergents TX-100 and NP-40, inhibition of photoaffinity labelling was seen at very low concentrations, and may reflect a specific interaction with P-glycoprotein. Neither of these amphiphiles shows toxicity in this concentration range (see Table I). Yet $CH^RB30$ cells are collaterally sensitive to TX-100 and NP-40, and CHO transfectants expressing the mouse *mdr1* gene product showed neither resistance nor sensitivity. In this regard, it is interesting that TX-100 in the 5–10 $\mu$M range greatly stimulated the ATPase activity of partially-purified P-glycoprotein in CHAPS solution (Doige *et al.*, 1993). It is possible that TX-100 and NP-40 interact with P-glycoprotein in a specific manner *via* hydrophobic association with transmembrane segments that are not part of the drug-binding site.

Further investigation of the phenomenon of collateral sensitivity revealed that it did not arise as a result of increased uptake of amphiphile into MDR cells. Association of TX-100 with intact cells was very rapid, and occurred by an unmediated mechanism. Increasing levels of drug resistance were found to be correlated with decreased association of $^3$H-TX-100 with the intact cell. This pattern is also characteristic of the association of MDR spectrum drugs with this cell line series, suggesting that these nonionic amphiphiles may be handled by MDR cells in the same manner as drugs to which the cells are resistant. Alternatively, this pattern might arise from a difference in the molecular nature of the plasma membrane, again as a result of P-glycoprotein overexpression. Flow cytometry and quantitative fluorescence studies using MC540, which is sensitive to the physical state of the bilayer, indicated that the lipid packing density in the outer leaflet of the plasma membrane is significantly increased in MDR cells, and this increase correlates with levels of drug resistance and P-glycoprotein expression. Tighter packing of the outer leaflet may restrict the partitioning of amphiphiles, causing the TX-100 uptake into the plasma membrane to be lower in MDR cells. Since MC540 is also known to be responsive to gross changes in membrane potential (Waggoner, 1985), it is possible that the differences in MC540 fluorescence intensity observed with increasing drug resistance reflect alterations in membrane potential. However, a change in membrane potential of 100 mV typically produces a fractional fluorescence change of 10% or less. Since changes in membrane potential of this magnitude within the series of MDR cell lines are unlikely, this possibility seems remote. Polarisation measurements using the fluorescence probe TMA-DPH showed no detectable differences in bulk membrane microviscosity between MDR and drug-sensitive cells. Either TMA-DPH is not sensitive to the particular alterations found in MDR cells, or MC540 is detecting a more local change in membrane structure within specific domains, rather than the average picture provided by TMA-DPH. Warren *et al.* (1992) recently showed that modification of the plasma membrane lipids of $CEM/VLB_{100}$ cells resulted in a dramatic reversal of the MDR phenotype, and suggested that changes in membrane packing or fluidity may result from insertion of P-glycoprotein into the plasma membrane. Our data support the proposal that P-glycoprotein overexpression produces changes in lipid packing, but not bulk microviscosity.

This work was supported by a grant to F.J. Sharom from the National Cancer Institute of Canada. The authors would like to thank Dr Victor Ling of the Ontario Cancer Institute for making the multidrug-resistant CHO cell lines available to us, and Dr Philippe Gros of McGill University for supplying the parent and *mdr1*-transfected LR73 cell lines.

**Abbreviations** BzAlk, benzalkonium chloride; CePyr, cetylpyridinium chloride; CHAPS, 3-[(3-cholamidopropyl)dimethylammonio]-1-propanesulfonate; CHO, Chinese hamster ovary; DBP, dibutylphthalate; DMSO, dimethylsulfoxide; DOC, sodium deoxycholate; DTAB, dodecyltrimethylammonium chloride; MC540, merocyanine 540; MeBz, methylbenzethonium chloride; MTT, 3-(4,5-dimethylthiazol-2-yl)-2,5-diphenyltetrazolium bromide; NP-40, Nonidet P-40; PBS, phosphate-buffered saline; SDS-PAGE, sodium dodecyl sulfate polyacrylamide gel electrophoresis; TMA-DPH, trimethylammonium-diphenylhexatriene; TOG, thiooctylglucoside; TX-100, Triton X-100.

### References

ARSENAULT, A.L., LING, V. & KARTNER, N. (1988). Altered plasma membrane ultrastructure in multidrug-resistant cells. *Biochim. Biophys. Acta*, **938**, 315–321.

BAKER, R.M. & LING, V. (1978). Membrane mutants of mammalian cells in culture. In *Methods in Membrane Biology*. Korn, E. (ed.), Plenum Press, New York, Vol. 9, pp. 337–384.

BECH-HANSEN, N.T., TILL, J.E. & LING, V. (1976). Pleiotropic phenotype of colchicine-resistant CHO cells: cross-resistance and collateral sensitivity. *J. Cell. Physiol.*, **88**, 23–32.

BRADFORD, M.M. (1976). A rapid and sensitive method for the quantitation of microgram quantities of protein using the principle of protein-dye binding. *Anal. Biochem.*, **72**, 248–254.

CARMICHAEL, J., DEGRAFF, W.G., GAZDAR, A.F., MINNA, J.D. & MITCHELL, J.B. (1987). Evaluation of a tetrazolium-based semiautomated colorimetric assay: assessment of chemosensitivity testing. *Cancer Res.*, **47**, 936–942.

CROOP, J.M., GUILD, B.C., GROS, P. & HOUSMAN, D.E. (1987). Genetics of multidrug resistance: relationship of a cloned gene to the complete multidrug resistant phenotype. *Cancer Res.*, **47**, 5982–5988.

DOIGE, C.A. & SHAROM, F.J. (1991). Strategies for purification of the P-glycroprotein from multidrug resistant Chinese hamster ovary cells. *Protein Expr. Purif.*, **2**, 256–265.

DOIGE, C.A., YU, X.-H. & SHAROM, F.J. (1992). ATPase activity of partially purified P-glycoprotein from multidrug-resistant Chinese hamster ovary cells. *Biochim. Biophys. Acta*, **1109**, 149–160.

DOIGE, C.A., YU, X.-H. & SHAROM, F.J. (1993). The effect of lipids and detergents on the ATPase activity of partially purified P-glycoprotein. *Biochim. Biophys. Acta*, **1146**, 65–72.

GEORGES, E., SHAROM, F.J. & LING, V. (1990). Multidrug resistance and chemosensitization: therapeutic implications for cancer chemotherapy. *Adv. Pharmacol.*, **21**, 185–220.

GREENBURGER, L.M., YANG., C.-P.H., GINDIN, E. & HORWITZ, S.B. (1990). Photoaffinity probes for the α-adrenergic receptor and the calcium channel bind to a common domain in P-glycoprotein. *J. Biol. Chem.*, **265**, 4394–4401.

GREENBERGER, L.M., LISANTI, C.J., SILVA, J.T. & HORWITZ, S.B. (1991). Domain mapping of the photoaffinity drug-binding site in P-glycoprotein encoded by mouse *mdr1b*. *J. Biol. Chem.*, **266**, 20744–20751.

GROS, P., BEN NERIAH, Y., CROOP, J.M. & HOUSMAN, D.E. (1986). Isolation and expression of a complementary DNA that confers multidrug resistance. *Nature*, **323**, 728–731.

GROS, P., DHIR, R., CROOP, J. & TALBOT, F. (1991). A single amino acid substitution strongly modulates the activity and substrate specificity of the mouse mdr1 and mdr3 drug efflux pumps. *Proc. Natl Acad. Sci. USA*, **88**, 7289–7293.

GROS, P., TALBOT, F., TANG-WAI, D., BIBI, E. & KABACK, H.R. (1992). Lipophilic cations: a group of model substrates for the multidrug resistance transporter. *Biochemistry*, **31**, 1992–1998.

HELENIUS, A. & SIMMONS, K. (1975). Solubilization of membranes by detergents. *Biochim. Biophys. Acta*, **415**, 29–79.

HIGGINS, C.F. & GOTTESMAN, M.M. (1992). Is the multidrug transporter a flippase? *Trends in Biochem. Sci.*, **17**, 18–21.

JURANKA, P.F., ZASTAWNY, R.L. & LING, Y. (1989). P-glycroprotein: multidrug resistance and a superfamily of membrane-associated transport proteins. *FASEB J.*, **3**, 2583–2592.

KESSEL, D. (1988). Probing membrane alterations associated with anthracycline resistance using fluorescent dyes. *Biochem. Pharmacol.*, **37**, 4253–4256.

KLOHS, W.D., STEINKAMPF, R.W., HAVLICK, M.J. & JACKSON, R.C. (1986). Resistance to anthrapyrazoles and anthracyclines in multidrug-resistant P388 murine leukemia cells: reversal by $Ca^{2+}$ blockers and calmodulin antagonists. *Cancer Res.*, **46**, 4352–4356.

LING, V. (1975). Drug resistance and membrane alterations in mutants of mammalian cells. *Can. J. Genet. Cytol.*, **17**, 503–515.

LING, V. & THOMPSON, L.H. (1974). Reduced permeability in CHO cells as a mechanism of resistance to colchicine. *J. Cell Physiol.*, **83**, 103–116.

LING, V., KARTNER, N., SUDO, T., SIMINOVITCH, L. & RIORDAN, J.R. (1983). Multidrug-resistance phenotype in Chinese hamster ovary cells. *Cancer Treat. Rep.*, **67**, 869–874.

LOE, D.W., GLOVER, J.R., HEAD, S. & SHAROM, F.J. (1989). Solubilization, characterization and detergent interactions of lymphocyte 5′-nucleotidase. *Biochem. Cell Biol.*, **67**, 214–223.

MOSMANN, T. (1983). Rapid colorimetric assay for cellular growth and survival: application to proliferation and cytotoxicity assays. *J. Immunol. Meth.*, **65**, 55–63.

PASTAN, I. & GOTTESMAN, M.M. (1991). Multidrug resistance. *Annu. Rev. Med.*, **42**, 277–286.

RAMU, A., GLAUBIGER, D., MAGRATH, I.T. & JOSHI, A. (1983). Plasma membrane lipid structural order in doxorubicin-sensitive and -resistant P388 cells. *Cancer Res.*, **43**, 5533–5537.

RAMU, A., RAMU, N. & GORODETSKY, R. (1991). Reduced ouabain-sensitive potassium entry as a possible mechanism of multiple drug resistance in P388 cells. *Biochem. Pharmacol.*, **42**, 1699–1704.

RAMU, A., RAMU, N. & ROSARIO, L.M. (1991). Circumvention of multidrug resistance in P388 cells is associated with a rise in the cellular content of phosphatidylcholine. *Biochem. Pharmacol.*, **41**, 1455–1461.

RIORDAN, J.R. & LING, V. (1985). Genetic and biochemical characterization of multidrug resistance. *Pharmacol. Ther.*, **28**, 51–75.

ROBERTSON, S.M., LING, V. & STANNERS, C.P. (1984). Co-amplification of double minute chromosomes, multiple drug resistance, and cell surface P-glycoprotein in DNA-mediated transformants of mouse cells. *Mol. Cell Biol.*, **4**, 500–506.

SAFA, A.R., GLOVER, C.J., SEWELL, J.L., MEYERS, M.B., BIEDLER, J.L. & FELSTED, R.L. (1987). Identification of the multidrug resistance-related membrane glycoprotein as an acceptor for calcium channel blockers. *J. Biol. Chem.*, **262**, 7884–7888.

SEHESTED, M., FRICHE, E., BUHL JENSEN, P. & DEMANT, E.J.F. (1992). Relationship of VP-16 to the classical multidrug resistance phenotype. *Cancer Res.*, **52**, 2874–2879.

SHEETZ, M.P. & SINGER, S.J. (1974). Biological membranes as bilayer couples. A molecular mechanism of drug-erythrocyte interactions. *Proc. Natl Acad. Sci. USA*, **71**, 4457–4461.

SIEGFRIED, J.A., KENNEDY, K.A., SARTORELLI, A.C. & TRITTON, T.R. (1983). The role of membranes in the mechanism of action of the antineoplastic agent adriamycin-Spin-labeling studies with chronically hypoxic and drug-resistant tumor cells. *J. Biol. Chem.*, **258**, 339–343.

TAMAI, I. & SAFA, A.R. (1991). Azidopine noncompetitively interacts with vinblastine and cyclosporine A binding to P-glycoprotein in multidrug resistant cells. *J. Biol. Chem.*, **266**, 16796–16800.

UEDA, K., CARDARELLI, C., GOTTESMAN, M.M. & PASTAN, I. (1987). Expression of a full-length cDNA for the human 'MDR1' gene confers resistance to colchicine, doxorubicin, and vinblastine. *Proc. Natl Acad. Sci. USA*, **84**, 3004–3008.

WAGGONER, A.S. (1985). Dye probes of cell, organelle, and vesicle membrane potentials. In *The Enzymes of Biological Membranes*, Martonosi, A.J. (ed). pp. 313–331. Plenum Press: New York and London.

WARREN, I., JARDILLIER, J.-C., MALARSKA, A. & AKELI, M.-G. (1992). Increased accumulation of drugs in multidrug-resistant cells induced by liposomes. *Cancer Res.* **52**, 3241–3245.

WHEELER, C., RADER, R. & KESSEL, D. (1982). Membrane alterations associated with progressive adriamycin resistance. *Biochem. Pharmacol.*, **31**, 2691–2693.

WILLIAMSON, P., MATTOCKS, K. & SCHLEGEL, R.A. (1983). Merocyanine 540, a fluorescent probe sensitive to lipid packing. *Biochim. Biophys. Acta*, **732**, 387–393.

YANG, C.-P.H., MELLADO, W, & HORWITZ, S.B. (1988). Azidopine photoaffinity labelling of multidrug resistance-associated glycoproteins. *Biochem. Pharmacol.*, **37**, 1417–1421.

YU, H. & HUI, S.-W. (1992). Merocyanine 540 as a probe to monitor the molecular packing of phosphatidylcholine: a monolayer epifluorescence microscopy and spectroscopy study. *Biochim. Biophys. Acta*, **1107**, 245–254.

ZAMORA, J.M., PEARCE, H.L. & BECK, W.T. (1988). Physical-chemical properties shared by compounds that modulate multidrug resistance in human leukemic cells. *Mol. Pharmacol.*, **33**, 454–462.